# Impairment between Oxidant and Antioxidant Systems: Short- and Long-term Implications for Athletes’ Health

**DOI:** 10.3390/nu11061353

**Published:** 2019-06-15

**Authors:** Cristina Nocella, Vittoria Cammisotto, Fabio Pigozzi, Paolo Borrione, Chiara Fossati, Alessandra D’Amico, Roberto Cangemi, Mariangela Peruzzi, Giuliana Gobbi, Evaristo Ettorre, Giacomo Frati, Elena Cavarretta, Roberto Carnevale, SMiLe Group

**Affiliations:** 1 Department of Internal Medicine and Medical Specialities, Sapienza University of Rome, 00161 Rome, Italy; cristina.nocella@uniroma1.it (C.N.); roberto.cangemi@uniroma1.it (R.C.); evaristo.ettorre@uniroma1.it (E.E.); 2 Department of General Surgery and Surgical Speciality Paride Stefanini, Sapienza University of Rome, 00161 Rome, Italy; vittoria.cammisotto@uniroma1.it; 3 Department of Movement, Human and Health Sciences, University of Rome "Foro Italico", 00135 Rome, Italy; fabio.pigozzi@uniroma4.it (F.P.); paolo.borrione@uniroma4.it (P.B.); chiara.fossati@uniroma4.it (C.F.); alessandamico@libero.it (A.D.); 4 Department of Medical-Surgical Sciences and Biotechnologies, Sapienza University of Rome, 04100 Latina, Italy; mariangela.peruzzi@uniroma1.it (M.P.); fraticello@inwind.it (G.F.); elena.cavarretta@uniroma1.it (E.C.); 5 Mediterranea Cardiocentro, 80122 Napoli, Italy; 6 Department of Biomedical, Biotechnological and Translational Sciences (S.Bi.Bi.T.), Anatomy and Histology Unit, University of Parma, Ospedale Maggiore, 43126 Parma, Italy; giuliana.gobbi@unipr.it; 7 Faculty of Medicine and Surgery, Course E, Sapienza University of Rome, 04100 Latina (LT), Italy

**Keywords:** oxidative stress, muscle damage, neurodegeneration, antioxidant, athletes

## Abstract

The role of oxidative stress, an imbalance between reactive oxygen species production (ROS) and antioxidants, has been described in several patho-physiological conditions, including cardiovascular, neurological diseases and cancer, thus impacting on individuals’ lifelong health. Diet, environmental pollution, and physical activity can play a significant role in the oxidative balance of an organism. Even if physical training has proved to be able to counteract the negative effects caused by free radicals and to provide many health benefits, it is also known that intensive physical activity induces oxidative stress, inflammation, and free radical-mediated muscle damage. Indeed, variations in type, intensity, and duration of exercise training can activate different patterns of oxidant–antioxidant balance leading to different responses in terms of molecular and cellular damage. The aim of the present review is to discuss (1) the role of oxidative status in athletes in relation to exercise training practice, (2) the implications for muscle damage, (3) the long-term effect for neurodegenerative disease manifestations, (4) the role of antioxidant supplementations in preventing oxidative damages.

## 1. Reactive Oxygen Species: The Patho-physiological Role

### 1.1. ROS-Mediated Cellular Signaling

Recently, there has been growing interest in reactive oxygen species (ROS), key cellular components that play an important role in various physiological conditions, as well as in the development of several diseases. ROS are small highly reactive chemical species that contain one or more unpaired electrons and are able to oxidize other compounds. ROS include both radical and non-radical oxygen-based molecules, such as hydroxyl radical (OH^▪^), hydrogen peroxide (H_2_O_2_), singlet oxygen (O_2_), and superoxide (O_2_^▪−^) [1].

The generation of cellular ROS is induced by both endogenous and exogenous stimuli; moreover, their production can derive both from enzymatic and non-enzymatic sources. ROS are mainly released as by-products or waste products of the mitochondrial oxidative metabolism. They are also released in other physiological and necessary reactions as a cellular defense mechanism in response to xenobiotics, cytokines production, and bacterial invasion [2]. The endogenous sources of ROS include different cellular organs such as mitochondria, peroxisomes, and endoplasmic reticulum (RE). In particular, mitochondria represent the main source of ROS generation via the mitochondrial electron-transport system [3] and the RE is a cellular organelle that plays a key role in ROS production as its lumen represents a suitable oxidizing environment [4]. These cellular sub-units produce ROS due to the presence of soluble and membrane enzymes, such as xanthine oxidoreductase (XOR), nitric oxide (NO) synthase, cytochrome P450 monoxygenase (P450s), lipoxygenase (LOX), and cyclooxygenase (COX) [5,6]. The membrane enzyme NADPH oxidase is a multimeric protein that catalyzes the reduction of an electron from O_2_ to O_2_^▪−^ and has a specific function in defense of the organism against invading microorganisms [7]. Finally, ROS-inducing agents, like radiation, pollutants, and exposure to nanomaterials represent extracellular sources of ROS generation [7].

ROS are second messengers involved in the determination of cell destiny and in modulation of various signaling pathways, for example, growth, differentiation, progression, and cell death [8]. Many studies showed that ROS could influence cell-signaling through modulation of transcription, activation of transcription, and activation of nuclear factor k B (NF-kB), Kelch-like-ECH-associated protein 1 (Keap1)-nuclear factor (erythroid-derived 2) like (Nrf2)-ARE and phosphoinositide-3-kinase (PI3K)-Akt signaling, as well as mitogen activated protein kinase (MAPK) cascades [9].

### 1.2. Endogenous and Exogenous Antioxidants 

Antioxidant systems play an important role in maintaining redox balance. Among the main endogenous antioxidants that participate in various physiological and pathological processes, there are antioxidant proteins, which have enzymatic activity, such as superoxide dismutase (SOD), catalase (CAT), and glutathione peroxidase (GPx) and non-enzymatic antioxidants, which are molecules capable of rapidly neutralizing ROS, like glutathione, lipoic acid, bilirubin, ferritin [10,11].

Exogenous antioxidants derive from the dietary sources [12], which are present in commonly consumed fruits, vegetables, beverages (juices, tea, coffee), nuts, and cereal products [13].

Antioxidants from the diet such as vitamin E, vitamin C, carotenoids, some minerals (Zn, Mn, Cu, Se) and polyphenols (flavonoids, phenolic acids, stilbenes, lignans) impact the activity of endogenous antioxidants by which they exert a synergistic effect to maintain redox homeostasis [14]. Many epidemiological and interventional studies suggested that it is possible to counteract the onset of pathological processes with the provision of antioxidant-rich diets. Thus, the organism could be protected against oxidative stress by consumption of antioxidant nutrients, namely vitamins C and E, carotenoids, and flavonoids, which would interfere with oxidative damage to the DNA, proteins, and lipids [15].

Indeed, more attention has been directed to exogenous antioxidants that, together with endogenous antioxidants, provide an important mechanism to prevent stress-derived damage to cells and tissues mediated by ROS. These exogenous molecules can be used to reduce oxidative stress-mediated cardiovascular risk and therefore, can represent a useful tool in clinical practice. Specifically, natural extracts, such as polyphenols, exert an antioxidant activity that include suppression of ROS formation by either inhibition of enzymes involved in their production, like NOX2 [16], scavenging of ROS [17], or upregulation or protection of antioxidant defenses [18].

### 1.3. Oxidative Stress and Correlated Diseases 

Oxidative stress occurs when there is an imbalance between ROS formation and the antioxidant defense systems in favor of pro-oxidant processes [19]. Indeed, one of the most important concepts in human physiology is redox homeostasis that plays a key role in cellular physiological and pathological events.

When oxidative stress persists, the excessive ROS accumulation influences many cellular signaling pathways with oxidative stress itself representing a common patho-physiological mechanism underlying many chronic diseases, such as cancer, cardiovascular diseases (CVDs), and neurodegenerative diseases (NDDs) [20,21,22].

It has been widely demonstrated that DNA oxidative damage is closely associated with cancer development because chromosomal defects and activation of oncogenes are induced by free radicals [23]. An increase in ROS levels has been emphasized in the development and progression of CVDs. In this context, overproduction of ROS mediates different signaling pathways able to promote vascular alteration, lesions development and progression, and consequently atherosclerotic plaque formation [24]. Furthermore, oxidative damage can be involved in the pathogenesis of NDDs that are commonly late-onset disorders, such as Alzheimer’s disease (AD), Parkinson’s disease (PD), Huntington’s disease (HD), and Amyotrophic lateral sclerosis (ALS) [25,26,27,28]. ROS overproduction in these NDDs leads to NF-κB activation and translocation to the cell nucleus where it drives the transcription of cytokines, chemokines, NO, prostaglandins, and leukocyte adhesion molecules. This favors proliferation, inflammation, invasion, and cell survival. The ultimate results are neurotoxicity and cognitive impairment [29].

Besides its well-defined role in manifested diseases, oxidative stress has been more recently presumed to change in the “pre-clinical stages of disease” among apparently healthy people. A variety of lifestyle factors such as exercise have also been identified as possible modulators of ROS-mediated oxidative stress predisposing to tissue damage or other diseases.

## 2. The Physiological and Pathological Role of Oxidative Stress in Physical Exercise

It has consistently been shown that physical exercise produces ROS. The production of ROS induced by physical exercise is an important signalling pathway for inducing biological adaptations to training. On the other hand, ROS production could also have a deleterious impact on cells and tissues, by inducing lipid and protein peroxidation [30]. The beneficial or detrimental effect of exercise-induced oxidative stress is dependent upon the ROS concentration, duration of exposure, and training status of the individual.

ROS, within physiological concentrations, are important signalling molecules that regulate growth, proliferation, and differentiation. They are also responsible for some key adaptations to exercise at the tissue and cellular levels. Specifically, ROS can regulate antioxidant systems by increasing the contents and activities of the main antioxidant systems expressed in muscle cells such as SOD1, SOD2, GPx, and CAT and then decreasing ROS concentration [31]. Moreover, ROS can stimulate the mitochondrial biogenesis cascade in response to endurance exercise [32]. Finally, ROS can play an important role as a stimulator for exercise-mediated skeletal muscle glucose uptake [33], showing a possible mechanism of enhanced insulin sensitivity in response to endurance exercise.

Conversely, when exhaustive exercise leads to a strong increase in ROS, which cannot be challenged by endogenous antioxidants, severe oxidative damage, including muscle weakness and fatigue, DNA mutations, lipid peroxidation, mitochondrial dysfunction, and apoptosis/necrosis occur. When muscle is injured, this leads to the activation of the neutrophils and macrophages via cytokines production. These immune cells, in turn, can excessively produce ROS along with endothelial cell amplifying oxidative damage.

As different levels of oxidative stress depend on the amount, frequency, and type of physical training, the role of oxidative stress in physical exercise could be evaluated by dividing subjects into three different categories: amateur athletes (1–5 hours/week), elite athletes (>5 hours/week), and master athletes (>5 hours/week, >35 years). Performance characteristics that distinguish the elite from the non-elite athletes include (1) running economy (efficiency), defined as the steady-state of submaximal oxygen uptake at a given running velocity; (2) anaerobic threshold, defined as the oxygen consumption during exercise above which aerobic energy production is supplemented by anaerobic mechanisms; and (3) VO_2max_, defined as the maximum integrated capacity of the pulmonary, cardiovascular, and muscular systems to uptake, transport, and utilize O_2_ [34]. Several human studies analysed the effects of different types of sports on oxidative stress, inflammation, and muscle damage according to several variables. A summary of the studies mentioned in this section is presented in Table 1. 

### 2.1. Oxidative Stress in Amateur Trainers

Physical exercise is known to increase ROS production that is essential to promote an adaptive response, together with multi-protein pathways and signaling [35]. The exercise-related production of ROS is characterized by the hormesis curve, a bell-shaped or an inverted U-shape curve, where the peak corresponds to the optimal zone of better tolerance against stressors and a larger range between the optimal zone and the functional end-points corresponds to a greater adaptive capability and tolerance [36]. Moderate physical activity reduces the incidence of oxidative stress-based diseases by stimulating antioxidant defense systems, such as SOD, CAT, GPx, and glutathione reductase (GR). This can be explained considering the different amount of ROS produced during moderate exercise, which act as mild stimulating stressors able to trigger a response falling into the optimal zone of the hormesis curve. On the contrary, exercise above an individual threshold can cause a maladaptive response, called overtraining. Therefore, the functional endpoints of the hormesis curve are physical inactivity and the overtraining [36]. A single bout of exhaustive exercise on the treadmill showed that Trolox-equivalent antioxidant capacity (TEAC) does not vary before and after the maximal effort in non-professional regular runners, while it is reduced in untrained sedentary subjects [37]. However, TEAC, a measure of the total antioxidant capacity, was substantially higher in trained individuals compared to untrained subjects due to the adaptive response. Moreover, the authors [37] demonstrated a correlation between the anaerobic threshold (AT) and thiobarbituric acid-reactive substances (TBARS), degradation products of lipid peroxidation and an indirect measure of ROS production. TBARS increased slightly in untrained subjects and decreased in trained runners, showing a negative correlation with AT, which is enhanced by training, thus ameliorating the tolerance to lactic acid and ammonia elimination. Accordingly, Seifi-skishahr et al. [38] compared the levels of plasma-reduced glutathione to oxidized glutathione (GSH/GSSG) and GSH/GSSG ratio in red blood cells in well-trained (WT), moderately trained (MT), and untrained (UT) subjects and found that 30 min after exercise the MT group showed the highest GSH/GSSG ratio, a redox biomarker, while the lowest GSH/GSSG ratio was recorded in the WT group [38]. In conclusion, long-term regular and moderate practice of aerobic physical activity protects against oxidative stress due to the adaptive response and favors the production of ROS and anti-oxidant enzymes, without causing significant damage to macromolecules, thus enhancing the tolerance of ROS without significant loss of function, in an exercise-mediated pre-condition via ROS.

### 2.2. Oxidative Stress and Elite Athletes

Even if the distinction between elite and non-elite athletes is not clearly defined in terms of exposure to physical exercise, an elite athlete is currently or has competed as a varsity player, a professional or a national/international level player. In elite athletes, the prolonged and intense exposure to physical activity may have the side effect of increased ROS production. In addition to training time, another important factor in ROS production is the type of exercise: aerobic exercise tends to increase peroxides production due to increased oxygen consumption, while anaerobic exercise produces less peroxides [39]. The body reacts and adapts in different ways depending on the type of exercise accomplished, as shown by a study conducted on female water polo and football players (compared to a control group). In this study, players who competed in water polo showed significantly lower O_2_^▪−^ levels compared to the control group and most importantly, compared to football players. Other by-products of physical activity (such as hydrogen peroxide and nitrites) are higher in athletes than in the control sedentary women group; however, athletes showed better antioxidant systems [40]. Other studies highlighted other important variables that influence ROS production and antioxidant defense systems such as duration, severity, and intensity of exercise [41,42,43], age [39], training status [44,45,46], and dietary intake [47]. For instance, in a comparison between basketball and soccer adolescent athletes, it has been highlighted that the former showed higher levels of total serum ROS compared to the latter; this information could be misleading, knowing that basketball is composed of a higher percentage of anaerobic exercise time compared to soccer (60% vs. 20% anaerobic, respectively): this can be justified considering the differences between soccer and basketball, the latter featuring shorter games with numerous intervals and pauses, which might alleviate the effect of anaerobic exercise on oxidative status. Even amongst professional players, such as handball players, oxidation biomarkers variate in response to the type of training performed (off-season, pre-season, early-season, and play-off), suggesting that ROS production does not depend on the sport itself but rather on the activity performed and the subsequent adaptation of the body: trainings and activities performed in early-stage and in play-off, which are significantly more intense than off- and pre-stage preparations, see an increase in oxidants production compensated by a higher production of antioxidant substances. In professional soccer players, deep redox homeostasis impairments were present among the soccer season, in particular at the beginning of the season compared to mid- and end-season [48]. Cavarretta et al. [49] performed a randomized clinical trial on 24 young elite male soccer players during the first month of the regular season demonstrating that compared to sedentary controls, elite soccer players showed lower anti-oxidant power and higher oxidative stress in terms of soluble NADPH oxidase 2 derived peptide (sNox2-dp), H_2_O_2_ production, and H_2_O_2_ breakdown activity (HBA), but these effects could be partially counteracted by dark chocolate administration, as antioxidant supplement.

Even if a wide variety of exercise protocols and assay procedures have been used to study oxidative stress pertaining to anaerobic work, these studies confirmed that high-intensity physical exercise can cause redox imbalance overwhelming the antioxidant defence ability, leading to several types of injuries.

### 2.3. Oxidative Stress and Master Athletes

Masters athletes are typically defined as individuals >35 years who either systematically train for or compete in athletic sport specifically designed for older adults at high levels despite the aging process. Research evidences indicate that aging increases the incidence of muscle injury and reduces muscle capacity that could potentially enhance oxidative damage [50]. However, several evidences showed that long-term endurance training might potentially reduce exercise-induced oxidative stress. A trend toward higher glutathione peroxidase enzymatic activity that was observed in amateur endurance master athletes, routinely practicing cycling, was an improvement in oxidative stress response that might preserve muscle mass [51]. Recent studies [52,53] were conducted on middle-aged master swimmers and long-distance runners and showed the effect of both 8-week high-intensity discontinuous training (HIDT) and “traditional” continuous moderate-intensity training (MOD) on oxidative damage. TBARS levels were significantly reduced in the HIDT group only after completion of the 8-week training program and not at mid-term. It is plausible that during the full duration of the training the antioxidant systems had enough time to repair damages caused by the single high-intensity training sessions. The MOD control group values, instead, show decreased values in TBARS even after 4 weeks of exercises. Protein carbonyls (PC) levels remain the same between the two groups, and markers of DNA damage (8-hydroxy-2-deoxy guanosine, 8-OHdG) levels are reduced post-training. Key difference between the two groups, however, is the total antioxidant capacity (TAC) levels: while in MOD trained subjects it is reduced, in HIDT it remains at pre-training levels. Furthermore, HIDT proved to be beneficial in master subjects trained with MOD: a 6-weeks long HIDT training period increased V’O_2_ peak by 12% and antioxidant capacity by 13%, with a significant decrease in baseline ROS production (−20%) compared to MOD in response to a physical stress test.

More recently, the effect of long-term endurance training was also confirmed in master endurance runners (ER) [54]. These athletes had better antioxidant/pro-oxidant ratios with lower values for redox parameters (TEAC/TBARS, SOD/TBARS, and CAT/TBARS) and higher NO levels. The mechanism that the authors proposed is the effect of long-term endurance training on telomere length maintenance. Indeed, ER runners have longer telomeres than age-matched controls, which in turn may be related to better NO bioavailability and redox balance status [54]

## 3. Muscle Damage in Athletes Induced by Redox Imbalance during Intensive Exercise

It has been widely demonstrated that both aerobic and anaerobic exercise lead to oxidative stress [55]. The first human trial confirming this theory was conducted by Dillard et al. in 1978 [56]. To date, a number of studies had reported findings concerning physical exercise-related ROS production and their role in muscle damage. Specifically, the exercise-induced muscle damage occurs in two different phases. The first consists in muscle damage during exercise and depends on several factors, all related to muscle fibers structure. Sarcomeres have a fundamental role in this process. Indeed, studies have demonstrated that dissimilarities in sarcomere length are triggered by coding genes polymorphisms [57,58,59] and, as a consequence, some types of sarcomeres can withstand better eccentric actions than others.

The second phase is linked to the delayed inflammatory response. Fibers that have been damaged cause leucocytes infiltration in the site of injury. This determines a sequence of effects including neutrophils contribution to the degradation of damaged muscle tissues by producing ROS, which in turn attract macrophages to the area of trauma [60]. This process modulates muscle remodeling; in an extreme case of muscle damage, remodeling may become maladaptive, characterized by necrosis, incomplete healing, and fibrotic scar tissue formation [61]. However, other research had also underlined that, depending on their concentration in the blood stream, ROS could also have positive effects [62]. If ROS level stands in the physiological range, they have a positive feedback on antioxidant production. It means that low-levels of ROS play a key role in exercise-induced adaptation of muscle phenotype. On the other hand, if ROS concentrations are too high, muscle tissues response become maladaptive with harmful consequences such as weakness, fatigue, DNA mutations, lipid peroxidation, mitochondrial dysfunctions, and cells apoptosis or necrosis as above reported.

ROS have both positive and negative effects depending upon several factors such as ROS concentration, duration of exposure, and training status of individuals as persons who are trained have higher levels of adaptation and fewer health risks. An important factor determining the role of increased ROS in muscle damage is represented by gene polymorphisms [63,64]. Even though the exact number of genes involved in physical activity and sport performance is constantly increasing due to new research and findings, approximately 165 autosomal genes, 5 on the X chromosome, and 17 mitochondrial genes have been identified.

Genes have a different expression because of different interaction between the individual and the environment: the biological outcome of different groups is hardly comparable because too many variables might change the results of studies.

Genetic background could influence oxidative response, with particular regard to the role played by single nucleotide polymorphisms (SNP). Indeed, it is becoming more and more important to study how genetic variants are involved in physical activity and how they could influence the physical quality, performance, and skill. Several SNPs have been identified as implicated in the dysfunction of the antioxidant mechanism with a consequential increase of the muscle damage by different mechanisms.

For example, SOD2 gene allocated on chromosome 6(6q25.3) codes for manganese superoxide dismutase (MnSOD), which catalyzes superoxide dismutation in mitochondria by converting anion superoxide into hydrogen peroxide and oxygen. Therefore, the inhibition of MnSOD activity causes the accumulation of ROS and leads to free radical-mediated damage to mitochondrial membranes and the apoptosis of cells. The T allele of the Ala16Val (rs4880 C/T) polymorphism in the mitochondrial SOD2 gene has been reported to reduce SOD2 efficiency against oxidative stress. Athletes with SOD2 TT genotype have increased creatine kinase (CK) value [65,66], creatinine levels [66], and increased advanced oxidation protein products (AOPP), lactic dehydrogenase (LDH), and myoglobin plasma levels; therefore it could be unfavorable for poor and straight performances in sport.

In addition to SOD2, polymorphisms of genes with antioxidant function have also an effect on muscle damage. CAT is a heme-dependent enzyme, localized in peroxisome, this gene is allocated on chromosome 11 (11p13) which catalyzes the breakdown of H_2_O_2_ to H_2_O and O_2_ with an extremely high turnover rate. GPx, is a Selenium-dependent enzyme and the respective gene is located on chromosome 3 (3p21.31); it reduces H_2_O_2_ or organic peroxides (ROOH) to H_2_O or alcohol (ROH), respectively. Vecchio et al. found that in water polo players having either CAT −844 GA or GPx1 CT genotype showed a significant increase in post-exercise oxidative stress and, respectively, GPx and CAT enzyme [77].

Other mechanisms potentially involved are related to the alteration of the inflammatory response generated in order to protect muscle from ROS overload.

For example, cytokines have an important role in the modulation and activation of the inflammatory response. The interleukin-1 (IL1) family of cytokines genes includes IL1A and IL1B and the receptor antagonist IL1Ra encoding by IL1RN gene, localized to the long arm of chromosome 2 at band 2q14.2. The absence of IL1RN leads to an increase in inflammatory response [78]. In vitro studies have shown that the IL1RN allele 2 is associated with decrease in IL1Ra and a moderate increase of pro-inflammatory phenotype, which negatively influences muscle remodeling and damage recovering [79].

Insulin-like growth factors (IGF-1 and IGF-2) influence the muscle remodeling process, since they have been proved to be crucial in the regeneration of the muscle. Different IGF-1 isoforms including IGF-1Eb and IGF-1Ec derived from alternative splicing process contribute as mechano-growth factors, since they are expressed when the muscle is overloaded or damaged. They also promote protection against ROS during the inflammatory response [80]. Every IGF2 SNPs studied are associated with a muscle strength loss, particularly in men, even if the link is still not completely clear [81].

Studies upon angiotensin 1 converting enzyme (ACE), encoded by a gene which is located on the long arm of chromosome 17 (17q23), and its ACE I/D polymorphisms suggest that allele I is associated with higher muscle damage. Angiotensin 2 is known to be involved in the inflammatory process following muscle damage. Blocking the angiotensin 2 receptor type 1 improves regeneration of injuries skeletal muscle and suppressed ROS production following strenuous exercise in mice [82].

## 4. Muscle Damage and Neurodegeneration in Athletes

Several studies reveal that retired professional athletes have a significantly higher probability of developing neurodegenerative diseases such as AD or Lou Gehrig’s disease, known as ALS [83]. Neurodegenerative diseases are characterized by the progressive loss of neuronal function or the death of neurons which lead to compromised motor or cognitive function [84]. The most common neurodegenerative diseases include AD, PD, HD, and ALS [85].

ALS is a neurodegenerative disease that can be caused both by genetic and environmental factors such as smoking; occupation; physical activity; and chemicals, such as heavy metals, ambient aromatic hydrocarbons, and pesticides [86,87]. Recently a growing number of studies support the role of excessive physical activity, increased ROS, and musculoskeletal trauma [88,89]. In fact, many sports, defined as contact-sports such as football, soccer, hockey, or boxing have also demonstrated a greater risk of developing ALS as associated with both vigorous physical activity and the risk of potential head and cervical spine trauma [89]. Moreover, among different hypotheses concerning the etiology and pathogenesis of ALS, oxidative stress is a well-established pathogenic mechanism. In 10 patients with ALS that performed an incremental bicycling test, the assessment of lipoperoxides and lactate during exercise showed that values were significantly higher in patients with ALS than in control subjects with various chronic neuropathies at rest, during exercise, and 30 min afterward. Moreover, the increase in lipoperoxide concentration during exercise strongly and positively correlated with lactate accumulation [90].

Besides this direct and primary effect, oxidative stress could also be a secondary event resulting from mechanical traumas. Indeed, during a traumatic brain and axonal injury, a perturbation of the cytoskeleton occurs, causing microtubule and neurofilament dissolution and pathologic reorganization of neurofilament proteins [91]. Trauma to the central nervous system (CNS) triggers stress responses that include oxidative stress due to ROS generation [92].

With this regard, an important study highlighted that there is a strong and highly significant relationship between being a professional soccer player and the development of ALS in a retrospective cohort study that included 7,325 male professional soccer players from the Italian First or Second Division in the period 1970–2001 [93]. However, the same results cannot be extended to other types of sports such as professional road cyclists and basketball players [94].

Moreover, in 2012 Lehman et al. investigated neurodegenerative causes of death in a contact sport such as football; in particular the study analyzed development of AD, PD, and ALS in a cohort of 3439 National Football League players. The results obtained showed neurodegenerative mortality of this cohort was three times higher than the general US population and specifically for AD and ALS it is four times higher [95]. Furthermore, another retrospective analysis in National Football League players showed a very high rate of ALS prevalence associated with professional football [96]. On the contrary, a case–control study explored the association between onset ALS and physical activities, with specific reference to trauma-related risk. They concluded that the practice of physical activity is not a risk factor for ALS per se but also sports-related microtraumas are involved as etio-pathogenic factors in the natural history of ALS [97]. A study highlights how trauma like spinal concussion, a spinal cord injury, could have a role in the genesis of ALS: the injury is caused by head collisions and interests the neck area and usually lasts for 24 hours among athletes [98]. However, studies have shown that longer-term effects of concussion could be a possible factor of genesis of ASL. Despite some cases report of athletes affected by ALS disease there is still not formal evidence of the associations of ALS and contact sports [99].

There are several causes that could lead to neurodegeneration in athletes, including genetic factors, oxidative stress, and even substance abuse as well as repetitive neurotrauma, which in some sports causes progressive changes in the microstructure and brain physiology. More researches are needed to better clarify the role and the consequences that these factors could have in the long term on neurological conditions in former athletes.

## 5. Impact of Antioxidant Supplementation in the Athlete Population

Currently, the evidences concerning any benefit or impairment on oxidative stress prevention in athletes and antioxidant supplementation are controversial [48]. It is adequately demonstrated that intense physical exercise is associated with a strong increase in oxidative stress in skeletal muscles [48], therefore the administration of antioxidant nutrients could represent a beneficial intervention to reduce the rate of oxidative stress-induced muscle injury in athletes. Interest in antioxidant supplements has been born among athletes and people who train regularly. For this reason, antioxidants are the most common supplements used by athletes both amateur and professional [100].

Among the studied antioxidant substances are C vitamin, E vitamin, Green Tea Extract (GTE), polyphenols, and N-acetyl-cysteine (NAC) [101,102,103,104]. The complexity in the comprehension of the effects increased as all these substances were administrated in different dosages, forms, and timing [105]. Effects of all these antioxidant substances have been studied on several disciplines able to induce oxidative stress. However the athletes recruited in the studies often practiced different sports at different levels, with the consequence of an accentuated heterogeneity of the results [106].

Major outcomes evaluated in trials ranged from simple performance measurements to more elaborate blood investigations like the presence of free radical species, endogenous (enzymatic) antioxidant power, muscular damage, and inflammatory markers [107].

Several human studies analysed the effects of supplementation with antioxidants in athletes. A summary of the studies mentioned in this review is presented in Table 2.

Regarding short term and high dosage C and E vitamin supplementation, acute effects on decreasing exercise-induced muscle damage and on lowering the inflammatory response during competition were achieved [106]. On the other hand, de Oliveira et al. showed that C and E vitamin supplementation reduced oxidative stress but did not attenuate markers of muscle damage or muscle soreness promoted by acute exercise and did not exert any ergogenic effect on football performance of young athletes [108].

Another group of natural antioxidants is polyphenols, which include a wide range of molecules found in many plant foods [109]. If, on the one hand, it has been demonstrated that polyphenols supplementation has a real ability in reducing the presence of free radical species, it is true that assured evidences on performance improvements and muscle damage markers reduction are yet inconsistent. As for this peculiar field of research, careful planning of future research will be required, in order to understand the effect that diet polyphenols have on specific selected markers [69].

Furthermore, one crossover experimental trial showed how GTE supplementation might have a potential in preventing physical activity induced oxidative stress without compromising endogenous antioxidant mechanisms. However, GTE supplementation does not prevent exercise induced muscle damage, nor improve performance, but supplementation with L-theanine, the main amino acid present in tea leaves, contributed to a significant post-exercise decrease in inflammation parameters such as IL-2, IL-10, and interferon (IFN)-γ and an improvement in the disturbed immune response in elite athletes [102,110]. Moreover, supplementation with GTE (500 mg/day) for 15 days in sixteen trained male amateur athletes showed an improvement in muscle damage and oxidative stress in response to fatigue as well as positive effects on neuromuscular function in response to a cumulative fatigue condition [111].

NAC supplementation showed several advantages. NAC intravenous administration showed short term improvements on athletes’ performance [112]. Two different studies reported that supplementation of NAC during a short period was effective to reduce oxidant action and increase antioxidant action whereas no alterations on cellular damage markers were obtained [18]. Moreover, Slattery and collaborators investigated oxidative damage and showed improvement of sprint performance, increase in post exercise antioxidant capacity, and reduction of exercise-induced oxidative damage finally, attenuation of inflammation was also observed [104]. NAC intravenous administration shows definite short-term improvements for athletes’ performance, yet intravenous supplements administration in athletes leads to an ethical debate on how much is considered correct as well as to potential anti-doping rule violations. As for oral administration of NAC, due to its hard management, further studies are needed [113].

If there are many studies that highlight the beneficial effects of a diet enriched with antioxidants, some researchers showed that there are some situations in which supplementation is probably disadvantageous [20,21,114]. In fact, what emerges from several studies deals with possible side effects that antioxidant substances supplementation might have on endogenous oxidative stress response mechanisms. Actually there must be a precise and correct balance between free radical species and antioxidant enzymes: if, on the one hand, free radical species may damage several cellular macromolecules (e.g., DNA and cell membrane constituents) [115], it is also true that their appearance, induced by physical activity, is considered necessary for several adaptations that eventually will lead to muscular angiogenesis and mitochondria synthesis. It has been observed that antioxidant supplementation may disturb the balance between free radical species and endogenous antioxidant mechanisms, altering the physiological adaptive responses [116]. Moreover, human studies reported a detrimental effect of antioxidant supplementation on exercise capacity, adaptive gene expression and protein synthesis, probably caused by an attenuation of the redox homeostasis pathway in muscle [114]. Recently, several authors have focused their attention to the study of personalized antioxidant interventions. In fact, the evaluation of specific antioxidant inadequacies or deficiencies could lead to individual antioxidant interventions that would improve physical performance. In particular, the methodology that involves personalized nutrition could represent the mechanisms by which the antioxidant state regulates human metabolism and performance [117].

In light of the studies analyzed and considering the dual role of ROS signaling, it is evident that lowering the levels of ROS could have a detrimental effect. Therefore, also the antioxidant supplementation should take into consideration the physiological levels of ROS. Then, it is necessary to optimize and tailor a specific antioxidant supplementation to the precise redox status and specific muscle damage or fatigue and according to a different kind of exercise for each individual.

## 6. Conclusions

The balance between ROS production and antioxidant systems is a very important condition for the life of organisms. In fact, ROS play a dual role: at low or moderate levels they have a beneficial action on cellular responses. In particular, moderate concentrations of ROS in vivo are regulatory mediators in signaling processes and responsible for restoring redox homeostasis modulating intracellular transduction pathways and transcriptional factors involved in cell proliferation, differentiation, and maturation [9].

Some authors speculated on the positive role of a transient high level of ROS induced by exercise. In fact, the ROS not only play an important role in regulating muscle contractile activity, but also promote muscle regeneration during muscle damage [131], improving insulin sensitivity [132], and vasodilation during exercise [133]. Furthermore, moderate ROS production is very important to regulate the nervous system, which regulates neuronal development. Redox signaling is required for neuronal cell expansion and proliferation [134]. ROS and oxidative states influence signaling cascades, important for neurogenesis by modulating the redox state of proteins such as protein kinase (PK)C or regulating transcriptional factors like the NF-kB [135].

**Figure 1:**
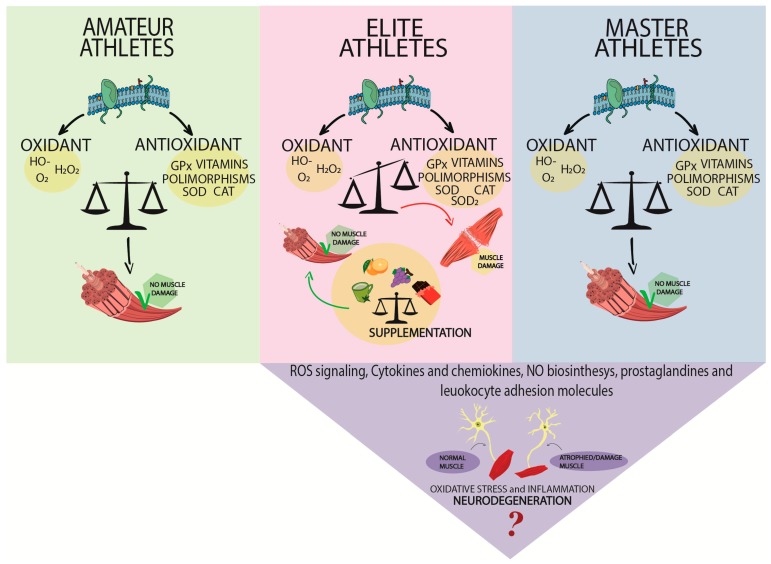
Schematic representation of the redox balance in three classes of athletes: amateur, elite, and master. High intensity exercise causes a redox imbalance that leads to different types of injuries, muscle damage, and development of neurodegenerative diseases. Furthermore, supplementation with antioxidants could restore the redox balance and reduce tissue damage mediated by oxidative stress in elite athletes.

Conversely, at high concentrations, they generate oxidative stress, a process that causes inflammation, oxidative damage to cells and tissues [136]. Recently, there is growing evidence that physical exercise is associated with oxidative stress-induced tissue damage. In particular, the analysis of the role of the oxidative state in three different categories of athletes such as amateur, elite, and master, highlighted that high-intensity physical exercise causes redox imbalance leading to several types of injuries and muscle damage. Moreover, this review showed that, among mechanisms implicated in the alteration of the redox balance, the presence of polymorphisms in antioxidant genes are associated with cellular damage. This injury leads on one hand to muscle damage, on the other hand to a greater risk of developing neurodegenerative diseases (Figure 1).

The implications of oxidative stress in the development of acute damage and in the predisposition to chronic-degenerative diseases suggest that antioxidant therapy could represent a promising strategy to reduce oxidative stress-mediated tissue damage in elite athletes or in other conditions associated with intensive physical exercise. Indeed, the data reported in this review indicate that antioxidant supplementation is associated with a beneficial impact on markers of oxidative stress, inflammation, and athletic performance. Although long-term studies are lacking, many interventional studies confirmed that intake of antioxidant substance has beneficial effects on inflammatory processes and chronic-degenerative diseases that are oxidative stress-mediated.

In conclusion, the development and improvement of training techniques focusing also on new nutrition/antioxidant supplementation strategies may help to reduce muscular damage and probably the risk of developing chronic-degenerative disease in elite athletes.

## Figures and Tables

**Table 1:** Effects of different types of sports on oxidative stress, inflammation, and muscle damage.

N of Subjects Male/Female Age (y)	Type and Time of Exercise	Type of Meal	Sampling	Effects	References
**AMATEURS**					
18 16/24 2.8 ± 1.4	Running/ 33.3 ± 0.5 h	Fluids (water, beverages and sports drinks) Carbohydrate-rich food (bread, fruits, cookies and pasta)	Serum and plasma 15 min and 48 h after the end of the race	TEAC ↑ TAC ↑ TBARS ↑ AT/TBARS ↓ PCC ↓ MDA ↑ 8-iso-PGF2a ↑ GSSG and GSSG/GSH ↑	[67]
23 23/0 60 ± 1.8	Cycling/ 8 times week	ND	Serum and plasma Baseline (vs. control group)	MDA ↓ GPx ↑ Antioxidant activity↑	[51]
33 33/0 42 ± 1.0	Treadmill test/ 7 stages (3 min each)	200 ml pear juice Two slices of bread	Serum Before and 30 min post exercise	TEAC ↔ TBARS ↓ GSH/GSSG ↑	[37]
10 10/0 21.1 ± 1.7	Treadmill test/ 35 min	Recorded normal diet	Plasma and serum Before, 10 min and 30 min post exercise	SOD ↑ CAT ↑	[38]
**ELITE**					
13 13/0 20.7 ± 2.5	Basket/ National League One Game	ND	Serum 40 min before and 45 min after game	TSP ↔ MPO ↔	[68]
35 35/0 21.6 ± 1.9	Soccer/ National League One Game	ND	Serum 40 min before and 45 min after game	TSP ↓ MPO ↔	[69]
22 22/0 26.5 ± 1.9	Soccer/ 15 min warmup and 90 min match	ND	Plasma Before and 5 min after training	Lactate↑ Glucose ↑ TAC↔ AST and ALT ↔ CK ↔ TBARS ↔	[70]
14 14/0 26.8 ± 1.2	Basket/ 1 regular season	ND	Plasma Before and after season (6 month, 59 matches)	TAC ↑↔ TBARS ↔ GSH ↓ CAT↔	[71]
33 33/0 17.1 ± 1.1	Handball/ Maximal progressive excercize test (bicyle ergometer)	ND	Plasma Post exercise test	SOD↑ CAT↓ VO_2_ max ↓ H_2_O_2_↑ NO_2_-↑	[72]
12 12/0 21.7 ± 6.0	Wrestlers	ND	Plasma Baseline (vs. baseline soccer and basketball players)	AOPP ↔ MDA ↔ TAC ↔	[73]
14 14/0 22.1 ± 4.4	Soccer	ND	Plasma Baseline (vs. baseline wrestlers and basketball players)	AOPP ↔ MDA↔ (vs. wrestlers), ↓ (vs. basketball) TAC ↔	[73]
13 13/0 20.2 ± 2.3	Basketball	ND	Plasma Baseline (vs. wrestlers and soccer players)	AOPP ↔ MDA↔ (vs. wrestlers), ↑ (vs. soccer) TAC ↔	[73]
15 0/15 20–23	Water polo	Dietary intake (2300-2400 Kcal/day)	Plasma and erythrocytes Baseline (vs. control group)	MDA ↑ TAS ↑ GSSG ↑ H_2_O_2_ ↑ O^2-^↑ SOD activity ↑ GPx ↔	[40]
19 0/19 20–23	Football				
10 10/0 25 ± 4.5	Handball/ Three training periods T2–T4 (8 week/each)	ND	Plasma, erythrocytes, neutrophils and lymphocyte Before, T2, T3, T4, after T4	TBARs ↑ Thiols ↑ CK ↑ lactate dehydrogenase ↑ aspartate aminotransferase↑ IL-6 and TNF-α ↑ uric acid ↑	[74]
14 14/0 25 ± 4.5	Handball/ Season match (60 min)	ND	Plasma, erythrocytes Before, after 60 min and after 24h	TBARs and Thiols ↑ Antioxidant activity ↑ GSH ↓ GSSG/GSH ↔ CK ↑ Lactate dehydrogenase ↑ IL-6 and TNF-α ↑ SOD ↑ CAT↓ GPx ↔	[75]
61 27/34 21.4 ± 1.6	Swimming/ High-intesity training session (3h, 4 weeks)	ND	Plasma, blood Before and after 4 weeks	GPx activity ↑ LPO ↑ GSSG/GSH ↑	[76]
**MASTER**					
16 16/0 30 ± 5	Swimming/ High intensity discontinous training	ND	Blood Before and after 6 weeks	ROS production ↑ Antioxidant capacity ↑	[52]
20 20/0 47.8 ± 7.8	Running/ High intensity discontinous training	ND	Plasma and urine Before and after exercise test	TBARS ↓ PC ↔ TAC ↓ 8-OH-dG ↓	[53]
10 10/0 51.6 ± 5.2	Endurance Races	ND	Plasma Baseline (vs. control groups)	TBARS ↔ TEAC ↓ SOD ↔ CAT ↔ NO^2-^ ↑ REDOX INDEX ↔ LTL ↓	[54]

Legend: the arrows represent increase (↑), decrease (↓), no change (↔). Abbreviation list: Trolox-equivalent antioxidant capacity (TEAC); total antioxidant capacity (TAC); thiobarbituric acid-reactive substances (TBARS), protein carbonyl content (PCC), anaerobic threshold (AT), malondialdehyde (MDA), glutathione peroxidase (GPx), glutathione reductase (GR), reduced glutathione (GSH), oxidized glutathione (GSSG), superoxide dismutase (SOD), catalase (CAT), total serum peroxides (TSP), myeloperoxidase (MPO), creatine kinase (CK), advanced oxidation protein products (AOPP), oxidation-reduction potential marker (sORP), lipid peroxidation (LPO), protein Carbonyls (PC), 8-hydroxy-2-deoxy guanosine (8-OH-dG), telomere length (LTL).

**Table 2:** Effects of antioxidant supplementation in Athletes. Main characteristics and main results of intervention studies.

	Treatment	Dose	Subjects N Kind of Sport	Study Duration	Markers	References
**1**	Vitamin C and Vitamin E	500 mg/d and 400 UI/d respectively	21 Football athletes	15 days	- MDA ↓ - Total lipid hydroperoxide ↓ - GSH/GSSH ↓ - FRAP ↓ - CK ↔ - VJH ↔ - Agility ↔ - Sprint test ↔ - Fatigue index ↔ - Muscle soreness ↔	[108]
**2**	L-theanine	300 mg/d	20 Rowing athletes	6 weeks	- IL-10 ↓ - IFN- γ ↑ - IL-2/IL-10 ↑ - IFN-γ/IL-10 ↑ - Th1/Th2 balance ↑ - CTL count ↓ - Treg/NK ↓ - Treg/ CTL ↓ - REP ↔	[110]
**3**	Dark Chocolate	40 g/d	24 young elite male football players 15 physically active male	30 days	- HBA ↔ - H_2_O_2_ ↓ - sNox2-dp ↔ - Myoglobin ↓ - CK ↓ - LDH ↓	[49]
**4**	N-acetyl-cysteine	1200 mg/d	20 Male volleyball athletes	14 days	- CK ↔ - AST ↔ - Creatinine ↓ - GPx ↔ - SOD ↔ - Glutathione ↓ - GSH ↔ - FRAP ↔ - LOOH ↔ - TBARS ↔	[118]
**5**	Green Tea Extract	500 mg/d	22 Healthy trained men	15 days	- CK ↓ - TBARS ↓ - Heart rate following exercise ↓ - EMG assessed neuromuscular electrical activity ↑	[111]
**6**	Vitamin C and Vitamin E	2000 mg/d and 1400 UI/d respectively	18 Elite Taekwondo athletes	4 days	- Myoglobin ↓ - CK ↓ - Heart rate following exercise ↔ - Blood lactate ↔ - Hemolysis ↓ - Plasma free radicals ↓	[106]
**7**	Quercetin Phytosome®	500 mg/d	48 Amateur Thriatlon athletes	2 weeks	- Training performance ↑ - Training efficacy ↑ - Post-run diffuse muscle pain ↓ - Cramps and localized pain ↓ - Recovery time ↓ - Plasma free radicals ↓	[119]
**8**	Grape Seed Extract	600 mg/d	40 Female Volleyball Players	8 weeks	- GSH ↑ - MDA ↓ - Serum insulin ↓ - HOMA-IR ↓ - CPK ↔ - TAC ↔ - NO ↔ - FPG ↔	[120]
**9**	Docosahexaenoic acid- and vitamin E	1 liter/d of isotonic beverage (278 mOsm/kg)	10 Young male Taekwondo athletes 8 Senior athletes	5 weeks	- Performance ↔ - Fatigue perception ↓ - Total polyphenol ↑ - MUFA and PUFA ↔ - MDA ↓ - Nitrotyrosine plasma levels ↓ - Antioxidant gene expression in PBMC ↔	[121]
**10**	Green Tea and Sour Tea (Hibiscus sabdariffa L.)	450 mg/d and 450 mg/d respectively	54 Male soccer player	6 weeks	- MDA ↓ - TAC ↑ - AST ↔ - CK ↔ - LDH ↔	[122]
**11**	Vitamin C, Vitamin A and Vitamin E	8 mg/kg/d, 16 ug/kg/d and 1 mg/kg/d respectively	14 Junior female figure skaters athletes	20 days	- HSPA1A gene expression ↓ - HSPB1 gene expression ↓	[123]
**12**	Chokeberry juice	150 ml/d	19 Rowing athletes	8 weeks	- IL-6 ↔ - TNF-α ↓ - TAC ↑ - UA ↔ - Myoglobin ↔	[124]
**13**	Green Tea Extract	980 mg/d	16 Sprinter athletes	8 weeks	- SOD ↓ - GPx ↔ - Total polyphenols ↑ - TAC ↔ - UA ↓ - MDA ↓ - CK ↔ - Lactate ↔ - Performance ↔	[102]
**14**	N-acetyl-cysteine	1200 mg/d	10 Male thriathletes	9 days	- Performance ↑ - TAC ↑ - TBARS ↓ - IL-6 ↓ - MPC-1 ↓ - NF-kB activity ↑	[125]
**15**	Resveratrol and Quercetin	120 mg/d and 225 mg/d respectively for 6 days; 240 mg/d and 450 mg/d respectively on day 7	14 Trained male adults	7 days	- F2-isoprostanes ↓ - FRAP ↔ - TEAC ↔ - ORAC ↔ - IL-8 ↔ - CRP ↔	[46]
**16**	Polyphenol-enriched protein powder (PSPC)	40 g/d of PSPC (2136 mg/d gallic acid equivalents)	38 Long distance runners	17 days	- CRP ↔ - Cytokines ↔	[125]
**17**	Quercetin and Vitamin C	500 mg/d and/or 250 mg/d respectively	60 Non-professional athletes	8 weeks	- IL-6 ↓ - CRP ↓ - E-selectin ↔ - F2-isoprostanes ↓	[126]
**18**	Quercetin and Vitamin C	500 mg/d and 200 mg/d respectively or 500 mg/d Quercetin or 500 mg/d Vitamin C	60 Non-professional athletes	8 weeks	- LDH ↓	[127]
**19**	Vitamin C and Vitamin E	250 mg/d and/or 400 UI/d respectively	64 Trained female athletes	4 weeks	- Performance ↔ - Myoglobin ↔	[128]
**20**	Coenzyme Q (10)	30 mg on day 1 90 mg on day 2 30 mg on day 3	20 Amateur running athletes	3 days	- IL-6 ↔ - TNF-α ↓ - GPx ↔ - H_2_O_2_ ↓ - CAT ↑ - TAS ↑ - Isoprostanes ↓ - 8-OHdG ↓	[129]
**21**	Flavanol-rich Lychee fruit extract	50 mg/d	20 Male long-distance runners	2 months	- Performance ↔ - NO ↔ - LDH ↔ - CPK ↔ - CRP ↔ - IL-6 ↔ - IL-10 ↔ - TGF-β ↑ - UA ↔ - ORAC ↔	[130]

Legend: the arrows represent increase (↑), decrease (↓), no change (↔). Abbreviation list: ferric reducing antioxidant power (FRAP); reduced glutathione (FRAP); oxidized glutathione (GSSH); malondialdehyde (MDA); vertical jump height (VJH); T-helper lymphocyte (Th); cytotoxic cells (CTL); natural killer (NK); interferon gamma (IFN-γ); T-regulatory lymphocytes (Tregs); interleukin-2 (IL-2); rowing ergometer performance (REP); hydrogen peroxide (H_2_O_2_); breakdown activity (HBA); soluble Nox2-derived peptide (sNox2-dp); lactate dehydrogenase (LDH); aspartate transaminase (AST); glutathione peroxidise (GPx); superoxide dismutase (SOD); thiobarbituric acid reactive substances (TBARS); lipid hydroperoxides (LOOH); electromyography (EMG); homeostasis model of assessment for insulin resistance 8 HOMA-IR); creatine phosphokinase (CPK); total antioxidant capacity (TAC); nitric oxide (NO); fasting plasma glucose (FPG); monounsaturated fatty acid (MUFA); polyunsaturated fatty acid (PUFA); peripheral blood mononuclear cells (PBMC); creatine kinase (CK); heat shock protein family A (Hsp70) member 1A (HSPA1A); heat shock protein family B (small) member 1 (HSPB1); interleukin-6 (IL-6); tumor necrosis factor alpha (TNF-α); uric acid (UA); monocyte chemotactic protein 1 (MPC-1); nuclear factor kappa B (NF-Kb); trolox equivalent antioxidant capacity (TEAC); oxygen radical absorptive capacity (ORAC); interleukin-8 (IL-8); C-reactive protein (CRP); high-density lipoprotein (HDL); catalase (CAT); total antioxidant status (TAS); 8-Hydroxy-20 -deoxyguanosine (8-OHdG); haemoglobin (Hb); interleukin-10 (IL-10); transforming growth factor beta (TGF-β).
